# Insights into the structural nature of the transition state in the Kir channel gating pathway

**DOI:** 10.4161/19336950.2014.962371

**Published:** 2014-10-30

**Authors:** Philip W Fowler, Murali K Bollepalli, Markus Rapedius, Ehsan Nematian-Ardestani, Lijun Shang, Mark SP Sansom, Stephen J Tucker, Thomas Baukrowitz

**Affiliations:** 1 Department of Biochemistry; University of Oxford, Oxford, UK; 2 Physiological Institute; Christian-Albrechts University; Kiel, Germany; 3 Clarendon Laboratory, Department of Physics; University of Oxford; Oxford; 4 OXION Ion Channel Initiative; University of Oxford; Oxford, UK; # Current address: School of Medical Sciences; Bradford University; Bradford, UK; § Current address: Department of Physiology; Development and Neuroscience; University of Cambridge; Cambridge, UK

## Abstract

In a previous study we identified an extensive gating network within the inwardly rectifying Kir1.1 (ROMK) channel by combining systematic scanning mutagenesis and functional analysis with structural models of the channel in the closed, pre-open and open states. This extensive network appeared to stabilize the open and pre-open states, but the network fragmented upon channel closure. In this study we have analyzed the gating kinetics of different mutations within key parts of this gating network. These results suggest that the structure of the transition state (TS), which connects the pre-open and closed states of the channel, more closely resembles the structure of the pre-open state. Furthermore, the G-loop, which occurs at the center of this extensive gating network, appears to become unstructured in the TS because mutations within this region have a ‘catalytic’ effect upon the channel gating kinetics.

## Introduction

Site-directed mutagenesis has become one of the most powerful and widely used strategies to probe structure-function relationships in ion channels. However, even with more recent atomic resolution structural information, it is often not immediately apparent how such mutations affect the equilibrium between the open and closed states of a channel. As a consequence, detailed mechanistic insight into the effects of these mutations on channel gating is limited to those rare cases where an identifiable interaction (e.g. a salt bridge or H-bond) is affected in a state-dependent manner. This difficulty arises because an individual mutation not only affects the immediate layer of directly interacting residues, but also tends to have more widespread ‘allosteric’ effects on other more distant residues.^1,2^ Insight into these more complex interactions only becomes possible if the gating effects of many residues (and ultimately all residues in the protein) can be determined and analyzed within the context of a structural gating pathway i.e. one which includes both the closed and open state conformations of the channel.

We have previously applied such an approach by systematically mutating the entire transmembrane/pore-domain of the pH-sensitive Kir1.1 (ROMK) channel (comprising mutations at over 190 positions).^3^ In that study, these data were then mapped onto models of Kir1.1 built using recently determined X-ray crystal structures of Kir channels in the closed,^4^ pre-open^5^ and open states,^6^ thereby reconstructing a possible gating pathway for the pH-dependent gating transitions of Kir1.1. Each mutation was then analyzed according to its impact on the intracellular pH at which half the maximal current is recorded (pH_0.5_ value). We assumed that a change in pH sensitivity was due to a change in the relative free energies of the open and closed states of the channel. Using this approach we identified 49 positions where mutations had a marked impact on the open-closed state equilibrium. More surprisingly, we found that 95% of these mutations increased channel pH sensitivity by destabilizing the open state more severely than the closed state. Subsequent mapping of these key residues onto homology models of Kir1.1 in the closed, pre-open and open states revealed that they assembled into distinct clusters of interacting residues and that the degree of clustering (i.e., the size of the largest cluster) was strikingly state-dependent.

In the closed state, these residues were fragmented into 5 physically distinct clusters: one formed by residues within the G-loop, and 4 identical clusters within the transmembrane domains (TMD). Upon movement into the pre-open state an upward movement of the cytoplasmic domain (CTD) causes the G-loop cluster to fuse with the 4 TMD clusters thereby creating a larger network of interacting residues, spreading throughout the core of the entire channel. Interestingly, further movements into the open state, associated with opening of the bundle-crossing gate, preserved the majority of these interactions. The greatest structural change in this gating network is therefore associated with movement from the closed to pre-open state and thus most likely represents the actual pH-sensitive gating conformational change that defines the measured pH_0.5_ value.

Based upon these observations, we proposed that the relative extent of network connectivity a residue experiences profoundly influences the thermodynamic effect of a point mutation on the open-closed state equilibrium, especially when the open and closed states differ so dramatically in their level of network connectivity. For example, when network connectivity is high (i.e. in the open and pre-open states of Kir1.1) then the structural perturbation caused by a single mutation can spread much further than it can in a state with low connectivity (i.e., in the closed state). Consequently, as observed in our systematic scan, most mutations preferentially destabilize the open state of the channel, resulting in an apparent increase in pH sensitivity.^3^

In this addendum to our previous study, we now characterize the impact of mutations on the kinetics of pH-gating to probe the structural nature of the rate-defining TS. Our results provide further insight into the structural basis of the Kir channel gating pathway and, in particular, the role of the G-loop.

## Results and Discussion

In our previous study we treated the pre-open conformation of the Kir channel as a structural intermediate of the pH gating pathway even though our functional analysis provided no direct evidence for (or against) its existence. Nevertheless, our previous mechanistic interpretations were not dependent upon the functional relevance of this state because the gating network was present and similar in both the open and pre-open states of the channel. By contrast, in this study, we assume that the pre-open state is functionally relevant. Furthermore, due to the structural (and presumably energetic) similarity of the pre-open and open states, we also assume that the transition state (which represents the energetically most unfavorable state in the reaction pathway) is located between the closed and pre-open states, not between the pre-open and open states.

**Figure 1.**
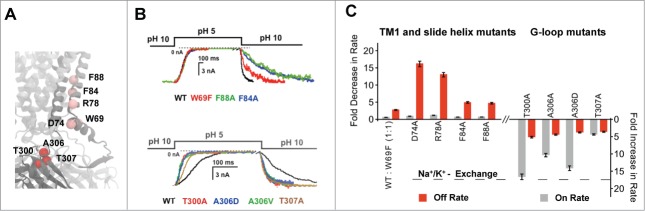
Mutations at several locations within the gating network differentially affect the pH-gating kinetics. (**A**) Location of the TM1, slide helix and G-loop mutations in the Open-Kir3.2 model. (**B**) Kinetics of pH-induced channel inhibition (pH 10 → pH 5) and pH recovery (pH 5 → pH 10) measured for WT and mutant channels. (**C**) Fold change in pH recovery (off-rate, red) and pH inhibition (on rate, gray) for TM1 and slide helix mutations (left), and G-loop mutations (right). The dotted line indicates the speed of the Na^+^ to K^+^ solution exchange for WT Kir1.1.

Based upon these assumptions, we used a fast piezo-controlled application system to induce rapid changes in internal pH to determine the kinetics of pH-inhibition; we used a rapid jump from pH 10 to pH 5 to measure the rate of inhibition (on rate), and vice versa to measure the rate of recovery (off rate). These rates were measured in giant excised patches for wild-type and mutant Kir1.1 channels expressed in Xenopus oocytes. We examined several mutations located in TM1 (F84A, F88A, R78A), the slide-helix (D74A, W69F), TM2 (A177V, I182A and K186A) and the G-loop (T300A, A306V, A306D, T307A) (**Fig. 1A**). All of these mutations are located within the previously characterized gating network and are therefore more sensitive to inhibition by increasing [H^+^] due to destabilisation of the more extensive gating network found in the open and pre-open states.

We found that the mutations in TM2 either had little effect (<2-fold change) on the gating kinetics (I182A and K186A), or, in the case of the A177V mutation, increased the rate of pH inhibition (≈9-fold) but slowed the recovery (≈4-fold). This suggests that these TM2 mutations affect all of the different gating states in the reaction pathway (including the TS) in a complex way that is difficult to interpret mechanistically and so they were not investigated further.

**Figure 2.**
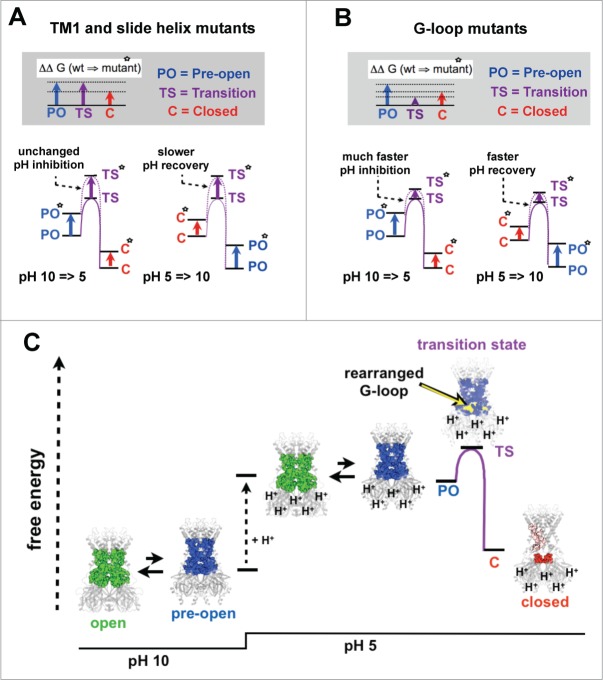
Gating kinetics provide insight into structure of the transition state. Free energy (ΔG) diagrams based on kinetic data for the closing (pH 10 → 5) and opening reactions (pH 5 → 10). These depict the energetic situation subsequent to the protonation and deprotonation steps. PO*, TS* and C* represent the relative free energies after point mutations are introduced in either TM1 and slide helix (**A**), or in the G-loop (**B**). Upper gray boxed panels indicate the change in free energy for the indicated states caused by the mutations; note that mutations in TM1 and slide helix (**A**) increase the free energy of the PO* and TS* states equally, but to a lesser extent the C* state (because the gating network is missing). This results in increased pH-sensitivity due to unchanged inhibition rates and slower activation rates. By contrast, G-loop mutations have relatively little effect on the TS* state (due to an unstructured G-loop). Instead, both the PO* and C* states are affected (**B**). However, the destabilising effect on the PO* state is greater than on the C* state leading to a faster rate of inhibition and thus an overall increased pH-sensitivity. (**C**) Cartoon depicting the structural and thermodynamic changes in Kir1.1 upon pH-inhibition. At pH 10, Kir1.1 exists predominantly in the open state (green residues indicate the gating network in this state). Protonation (pH 5) causes an increase in the free energy of the open state and pre-open states (blue residues indicate the gating network in this state) favoring the transition into the more stable closed state (red residues indicate the defragmented gating network). Kinetic analysis of mutants shown in **Figure 1** suggests that the structure of the rate determining transition state most closely resembles the pre-open state (gating network shown in blue), but with a different G-loop structure (yellow). This altered G-loop structure appears to be less sensitive to mutagenic destabilization than the open, pre-open or closed state.

Remarkably, however, the 5 mutations we measured within TM1 and the slide-helix had a strikingly similar gating phenotype. **Figure 1B and 1C** shows that they had little effect on the kinetics of pH-inhibition, but all markedly slowed the rate of recovery from pH-inhibition, consistent with their measured increase in pH-sensitivity. This indicates that these mutations had little effect on the energetic barrier between the pre-open state and the TS, but increased the barrier between closed state and the TS state (**Fig. 2A**). In other words, these mutations appear to have energetically destabilized both the pre-open state and TS to a similar extent, while only having a small effect on the closed state. The simplest structural interpretation of this finding is that the gating network remains mostly intact in the TS which therefore most closely resembles the pre-open state (**Fig. 2C**). This also suggests that the downward movement of the CTD (which fragments the network as the channel moves into the closed state) has not yet occurred.

By marked contrast, mutating the gating-network residues within the G-loop (T300A, A306V, A306D and T307A) had a ‘catalytic’ effect upon channel gating i.e. the mutations produced increases in both the rate of pH inhibition and the rate of recovery (**Fig. 1B and 1C**). However, their effect on the rate of inhibition was clearly more pronounced than their effect on the rate of recovery, and may also have even been underestimated due to the limitations of the speed at which the solutions can be exchanged. The more pronounced effect on speeding up the rate of pH-inhibition by the G-loop mutations is also consistent with their observed increase in pH-sensitivity, and thus, with the concept that the pre-open state is more strongly destabilised than the closed state by these mutations.

The faster gating kinetics of these G-loop mutations also indicates that they have less of a destabilizing effect on the TS compared to their effect on either the closed or pre-open states (**Fig. 2B**). Such a result can be explained if the G-loop becomes disordered or unstructured in the TS. Consequently, the mutations would have less of a destabilizing effect upon the G-loop in the TS if it is unstructured, compared to their effect on a more ordered G-loop structure in either the closed or pre-open states (**Fig. 2C**).

We would like to emphasize that the effect of these mutations on the rates of pH-inhibition and recovery provide a purely qualitative, rather than quantitative, link to their effects on the observed pH_0.5_ values; e.g., the F84A and F88A are markedly different in their pH_0.5_ values (7.4 ± 0.1 compared to 8.3 ± 0.1), but their effect on the rates of pH-inhibition and recovery are similar. It is therefore possible that such mutations may have additional indirect effects on the actual H^+^-sensing residue(s). Nevertheless, our mechanistic interpretation of these kinetic data appear justified because for the majority of the mutations we describe here and elsewhere,^7-9^ the relative effects on the rates of pH gating appears to explain the observed increase in pH sensitivity.

We therefore summarize our current mechanistic and structural view of pH-gating in Kir1.1 as follows (see also **Fig. 2**). Because the open-probability of Kir1.1 is relatively high at low [H^+^] (e.g., PO > 0.7 at pH 7.4)^10^ and the pre-open state is a functionally closed (non-conductive) state, we therefore assume that there is relatively little energetic difference between these 2 states and that they may interconvert frequently (possibly causing open state flicker). However, upon a rapid pH-jump to increased [H^+^] we assume that the titratable interactions which stabilize the open state are altered, thereby causing the free energy of the protonated open and pre-open states to rapidly increase relative to the closed state; this will cause the protonated channel to relax into the closed state by passing through the TS.

Our results suggest that the TS most closely resembles the pre-open state because the gating network remains mostly intact in this state. However, a structural change appears to occur within the G-loop of the TS. We propose that this change represents an unfolding or disordering of the G-loop because mutations within this region have a ‘catalytic’ effect on channel kinetics i.e., they speed up both the on and off rates of pH gating (**Fig. 1C**). Therefore the structural rearrangements we observe within the G-loop may represent a critical and rate-determining step during pH gating. Such rearrangements may also promote disengagement of the CTD from the TM/pore domain, thereby allowing it to move down/away and into the closed state conformation. Interestingly, many previous studies have also suggested that the flexibility and movement of the G-loop is critical for gating in several different Kir channels^11,12^ and may even act as a physical gate^13^(i.e. barrier to ion permeation).

In summary, our results provide a novel insight into the structural mechanisms which underlie the pH-dependent gating pathway of the Kir1.1 channel. Furthermore, the high conservation of structural architecture within the eukaryotic Kir channels suggests that the mechanisms we have determined for Kir1.1 are likely to be applicable to other members of this physiologically important family of potassium channels.

## Methods

Our methods for measurement of Kir1.1 pH-sensitivity and gating kinetics and constructing the models of Kir1.1 have all been described previously.^3,8^ Briefly; mutagenesis of the rat Kir1.1a in the pBF oocyte expression vector was performed using the QuikChangeII system (Agilent). mRNAs were synthesized using the SP6 mMESSAGE mMACHINE kit (Ambion). Manually defolliculated Xenopus oocytes were injected with 2–5 ng mRNA and the intracellular pH-sensitivity determined from giant patches in inside-out configuration under voltage-clamp conditions 3–7 d after mRNA injection. Pipettes were made from thick-walled borosilicate glass, had resistances of 0.3–0.9 MΩ (tip diameter of 5–15 μm) and filled with (in mM, pH adjusted to pH 7.2 with KOH) 120 KCl, 10 HEPES and 1.8 CaCl_2_. Currents were sampled at 1 kHz with an analog filter set to 3 kHz (-3 dB). Solutions were applied to excised patches via a piezo-driven fast application systems^8^ and had the following composition (in mM): 120 KCl, 10 HEPES, 2 K_2_EGTA, adjusted to the appropriate pH with HCl (pH 5) or KOH (pH 10). Models of Kir1.1 in both closed and pre-open conformations were primarily built using structures of Kir2.2^4,5^ while models of open Kir1.1 were built using a symmetrised open structure of Kir3.2^6^ as described previously.^3^
